# Desorption Electrospray Ionization Mass Spectrometry Imaging Techniques Depict a Reprogramming of Energy and Purine Metabolism in the Core Brain Regions of Chronic Social Defeat Stress Mice

**DOI:** 10.3390/metabo14050284

**Published:** 2024-05-15

**Authors:** Yulong Song, Fan Xiao, Jiye Aa, Guangji Wang

**Affiliations:** Key Lab of Drug Metabolism & Pharmacokinetics, State Key Laboratory of Natural Medicines, China Pharmaceutical University, Tongjiaxiang 24, Nanjing 210009, China; yulongsongnj@163.com (Y.S.); xianfengwunian@163.com (F.X.)

**Keywords:** spatial metabolomics, CSDS, depression, mass spectrometry imaging, purine metabolism, energy metabolism

## Abstract

Depression is associated with pathological changes and metabolic abnormalities in multiple brain regions. The simultaneous comprehensive and in situ detection of endogenous molecules in all brain regions is essential for a comprehensive understanding of depression pathology, which is described in this paper. A method based on desorption electrospray ionization mass spectrometry imaging (DESI-MSI) technology was developed to classify mouse brain regions using characteristic lipid molecules and to detect the metabolites in mouse brain tissue samples simultaneously. The results showed that characteristic lipid molecules can be used to clearly distinguish each subdivision of the mouse brain, and the accuracy of this method is higher than that of the conventional staining method. The cerebellar cortex, medial prefrontal cortex, hippocampus, striatum, nucleus accumbens-core, and nucleus accumbens-shell exhibited the most significant differences in the chronic social defeat stress model. An analysis of metabolic pathways revealed that 13 kinds of molecules related to energy metabolism and purine metabolism exhibited significant changes. A DESI-MSI method was developed for the detection of pathological brain sections. We found, for the first time, that there are characteristic changes in the energy metabolism in the cortex and purine metabolism in the striatum, which is highly important for obtaining a deeper and more comprehensive understanding of the pathology of depression and discovering regulatory targets.

## 1. Introduction

The mass spectrometry imaging technologies applied to biological samples can be divided into secondary ion mass spectrometry (SIMS) [1], matrix-assisted laser desorption ionization (MALDI) [2], and desorption electrospray ionization (DESI) [3]. SIMS is capable of detecting a wide range of elements, both metals and nonmetals, and can provide valuable information on the chemical bonds and molecules present on a sample surface [4]. However, due to the typically high energy of the primary ion beam, there is a risk of breaking covalent bonds and generating numerous fragment ions during testing. As a result, SIMS is generally more suitable for analyzing inorganic materials and those with surface chemical bonding, as well as for elemental analysis, inorganic analysis, and the detection of small molecules [5]. MALDI technology offers the advantage of high resolution [6] and has been extensively used to study substance variations across different regions in plant [7] and animal samples [8]. However, not all substances of interest can be effectively analyzed using a single matrix [9], which imposes certain limitations on substance identification. In contrast, DESI involves simpler sample pretreatment and obviates the need for matrix alteration during substance imaging [10], enabling comprehensive metabolomics research by facilitating the visualization of a broader range of substances [11]. The progression of DESI technology in recent years has enabled its increasing use in substance research pertaining to the brain [12] as well as pharmacodynamic evaluation of central nervous system (CNS) drugs [13,14].

Depression is a significant public health concern and a major contributor to the global disease burden. However, the underlying mechanisms of depression remain unclear. Previous studies have indicated that depression is closely associated with purine metabolism [15], energy-related metabolism [16,17], and amino acids [18,19]. Research on depression in various brain regions has been conducted. Notably, the medial prefrontal cortex (MPFC) of rats subjected to chronic social defeat stress (CSDS) exhibits substantial alterations [20], and inflammation or injury in the striatum also influences depressive symptoms [21,22]. Moreover, the nucleus accumbens [23] and the hippocampus [24,25] play crucial roles in depressive behavior. Recent investigations have revealed that previously overlooked brain regions, such as the cerebellum, also contribute to depression models [26,27]. Therefore, it is imperative to comprehensively investigate metabolic alterations across all regions of the plane. In this context, we employed DESI-MSI technology to elucidate the pivotal areas experiencing metabolic changes.

However, despite the availability of detailed classification methods for brain regions [28], the application of these methods often varies due to discrepancies between mass-spectrometry-based material distributions and anatomical structures [13,29]. Therefore, we aimed to establish a brain partitioning standard based on intrinsic differences rather than relying on traditional techniques such as HE staining [30] or optical imaging [31]. Under our experimental conditions, we were able to accurately detect relatively stable lipid profiles in the mouse brain. Moreover, significant variations were found among different regions, suggesting their potential utility for brain partitioning purposes. Consequently, we developed a DESI-MSI analysis method utilizing lipid profiling and investigated differentially abundant metabolites and their distinct distribution characteristics in the mouse brain in a CSDS-related depression model.

## 2. Materials and Methods

### 2.1. Chemicals and Reagents

Inosine, lactic acid, glucose, and glutamine were purchased from Sigma–Aldrich (St. Louis, MO, USA). Adenosine 5′-monophosphate (AMP), hypoxanthine, deoxyguanosine, uridine, fumaric acid, malic acid, aspartic acid, and dipotassium phosphate were obtained from Aladdin (Shanghai, China). Liquid chromatography-mass spectrometry (LC/MS)-grade acetonitrile was obtained from Merck (Darmstadt, Germany). Ultrapure water was generated using a Milli-Q system (Millipore, Bedford, MA, USA).

### 2.2. Animal Models of Depression

Male C57BL/6 J mice (6–7 weeks old) and CD1 aggressive mice (6–8 months old) were purchased from Vital River Experimental Animal Co., Ltd. (Beijing, China). The animals were housed in a controlled environment with a constant temperature, relative humidity of 60% ± 2%, and 12 h light/dark cycles. All mice were allowed free access to chow and water. Following a one-week acclimatization period, the C57BL/6 J mice underwent chronic social defeat stress (CSDS) model stimulation [32]. Briefly, C57BL/6J mice were exposed to daily 10 min aggressive attacks from CD1 mice for 10 days, with the duration gradually reduced by 0.5 min each day. Furthermore, each C57BL/6J mouse encountered a different CD1 mouse every day. Following the attacks, injured C57BL/6J mice were placed on the opposite side of a transparent divider in their CD1 mouse cage, allowing them 24 h of visual, olfactory, and auditory contact. Depressive behavior indicators including social interaction (SI) and the forced swimming test (FST) were evaluated according to the established literature protocols. The experimental protocol (no. 2021-09-038) was approved by the Animal Ethics Committee of China Pharmaceutical University (Nanjing, China).

### 2.3. Tissue Preparation

The mice were sacrificed with diethyl ether, and the brain tissue was immediately frozen with dry ice and stored at −80 °C. A small amount of gelatin was used to fix the brain tissue, and 15 μm thick sagittal slices were prepared using a cryostat microtome (Leica CM1950, Leica Microsystem, Wetzlar, Germany). After thawing, the slices were loaded onto positively charged microscope slides (Thermo Fisher Scientific, Waltham, MA, USA) and stored at −80 °C. To ensure consistency, the left side of the mouse brain was sliced 0.72 mm away from the sagittal suture to ensure that different samples were located in the same position. All samples were dried at −20 °C for 1 h and then placed at room temperature for 2 h for DESI-MSI analysis.

### 2.4. DESI-MSI Data Acquisition

DESI-MSI experiments were conducted using the DESI-XEVO-G2-XS-QTOF platform (Waters, Milford, MA, USA), and MSI analysis was performed in both positive and negative ion modes of the XEVO-G2-QTOF mass spectrometer. Prior to analysis, the DESI-MSI instrument was calibrated using a 0.5 mM sodium formate solution prepared in 90% 2-propanol. A Harvard Apparatus Pump 11 Elite (Harvard Apparatus, Holliston, MA, USA) was employed for delivering the spray solvent during data acquisition. Rhodamine 6G was utilized for optimizing DESI performance, with its signal strength remaining stable at e7 for over 10 s, indicating signal stability. Additionally, leucine enkephalin served as an internal standard for mass spectrum correction. The solvent flow rate was set at 2 μL/min, covering a mass range of 50–1000 Da with an ACN/H_2_O (8:2) solvent composition ratio while achieving lateral and longitudinal resolutions of 100 µm. Brain slices from CSDS mice and control mice were placed on the same slide to ensure consistent data acquisition across both sample sets.

### 2.5. DESI-MSI Data Analysis

The DESI-MSI data were acquired using Mass Lyn x 4.1 and HDI Imaging ver 1.6 software (Waters, Milford, MA, USA). TIC normalization was applied to the mass spectrometry data, which were then imaged in HDI and imported into Waters MSI Segmentation and Waters MSI Analyte Browser for UMAP dimensionality reduction. Progenesis QI was utilized for isotopic interference elimination and material identification. Multivariate data analysis was performed using SIMCA software (Sartorius Stedim Biotech, Umeå, Sweden, version 14.1), with PCA providing an overview of each brain region. Column charts were generated using GraphPad Prism (Version 8.0.1, La Jolla, CA, USA). Statistical analyses were conducted using SPSS software (version 19.0, IBM, Armonk, NY, USA), with *p*-values from Spearman’s and Pearson’s correlation adjusted using the FDR method at a significance threshold of *p* = 0.05. Heat maps were created using TBtools [33].

## 3. Results

### 3.1. Experimental Procedures and Lipid Partitioning of DESI-MSI

**Figure 1:**
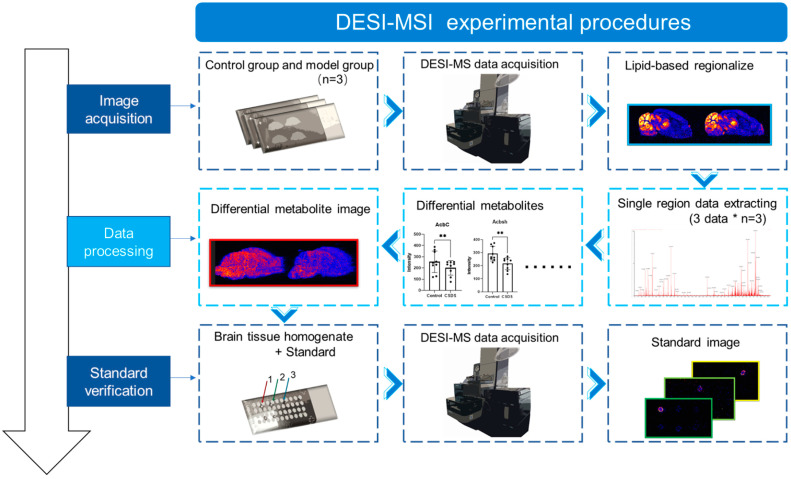
Workflow of DESI-MSI analysis of metabolic characteristics in the brains of CSDS depression model mice; *n* = 9 for statistical analysis. After conducting independent statistical analysis of 14 brain regions, we comprehensively validated and selected the differential metabolic mass spectrometry imaging maps. Mouse brain homogenates were added to the standard solution and compared with mouse brain homogenates to mitigate interference from the experimental matrix. *: *p* < 0.05, **: *p* < 0.01.

The complete experimental procedure is illustrated in Figure 1. The procedure included the imaging of the endogenous metabolites in the mouse brain, data analysis, and validation of the metabolite standards (Figure 1). For DESI-MSI imaging of mouse brains, three C57BL/6J mice each from the control group and the CSDS group were utilized. The brains of the mice were dissected, sectioned, and mounted on slides. Positive and negative ion signals were acquired using DESI-MSI, followed by data processing. After excluding the isotopic imaging results (Figure S1), the ions with optimal signal responses were selected for analysis of the spatial distribution of the metabolites (Figure S2). The analysis involved randomly sampling three data points from each brain region and summarizing three biological samples from each group. Statistical analysis was conducted with a sample size of *n* = 9. The corresponding mass spectrometry imaging maps were determined and verified according to the differentially abundant metabolites detected in the fourteen brain regions. For liquid samples, desiccation was performed to simulate substance determination under the experimental DESI conditions. The standard solution was continuously dripped onto various locations on the same slide while being sampled simultaneously to eliminate any interference from the other experimental procedures. During data processing, comparisons and analyses of the mass-charge ratios of the imaging results obtained from the standards and from the mouse brain images allowed for the further identification of the altered substances.

**Figure 2:**
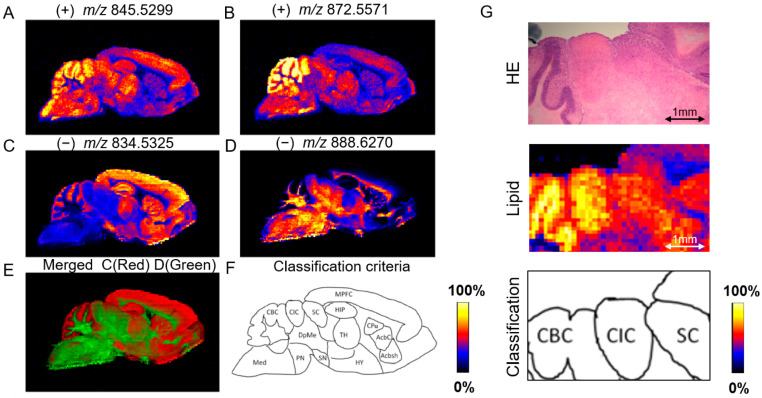
Classification of brain regions through clear lipid imaging. (**A**) Lipid 1, *m*/*z* 845.5299 in positive ion mode. (**B**) Lipid 2, *m*/*z* 872.5571 in positive ion mode. (**C**) Lipid 3, *m*/*z* 834.5325 in negative ion mode. (**D**) Lipid 4, *m*/*z* 888.6270 in negative ion mode. (**E**) Merged image: Lipid 3 is red, while Lipid 4 is green. (**F**) Results of regional classification based on lipid formation. (**G**) Comparison between lipid classification methods and the traditional HE classification method.

The optimized DESI conditions provided clear and accurate imaging of the lipid substances in the mouse brain, as depicted in Figure 2. Consequently, we proposed a novel classification method based on the in situ imaging lipid classification technique. As illustrated in Figure 2, both positive (Figure 2A: *m*/*z* 845.5299 and Figure 2B: *m*/*z* 872.5571) and negative (Figure 2C: *m*/*z* 834.5325 and Figure 2D: *m*/*z* 888.6270) ion modes provided clear and stable imaging results for the two lipids. Notably, a series of lipid mass spectrometry images revealed distinct and well-defined boundaries. Employing HDI software processing, different colors were assigned to represent corresponding mass-charge ratios at identical positions within the image (Figure 2E). Through this technique, we observed complete overlap of boundaries when various lipids were utilized for regional division. Hence, brain regions could be classified based on the distribution of lipid components. According to the analysis of the brain region imaging results, we defined 14 categories as criteria for classifying brain slices from different regions (Figure 2F): medial prefrontal cortex (MPFC), hippocampus (HIP), caudate putamen (striatum) (CPu), nucleus accumbens-core region (AcbC), nucleus accumbens-shell (Acbsh), thalamus (TH), hypothalamus (HY), central nucleus of the inferior colliculus (CIC), deep mesencephalic nucleus (DpMe), superior colliculus (SC), substantia nigra (SN), cerebellar cortex (CBC), pons (PN), and medulla (Med).

To validate the efficacy of the lipid classification system, we conducted a comparative analysis between the conventional HE staining method and the proposed lipid classification approach (Figure 2G). Notably, lipid mass spectrometry exhibited superior discriminatory power in distinguishing the CIC and SC brain regions. Furthermore, across the other areas depicted in Figure 2, the lipid classification method demonstrated enhanced adaptability for classifying diverse brain regions.

### 3.2. Significant Changes in 13 Metabolites Induced by the CSDS Model

**Figure 3:**
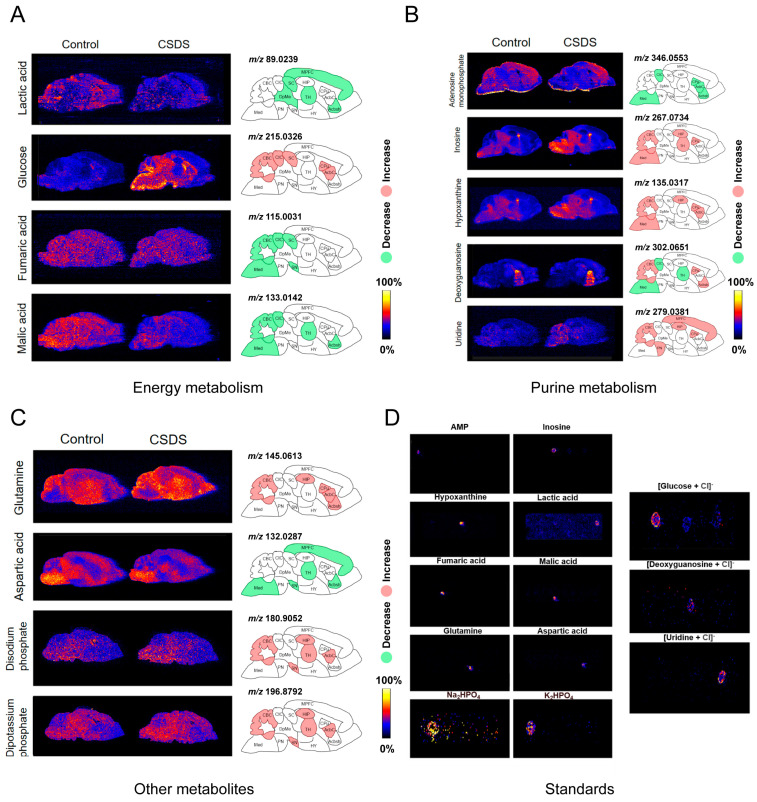
Thirteen differentially abundant metabolites and their corresponding substance validation. (**A**) Images of energy-metabolism-related metabolites and their most prominent brain regions. (**B**) Images of purine-metabolism-related metabolites and their most prominent brain regions. (**C**) Images of other metabolites and their most prominent brain regions. (**D**) Validation of standards for 13 metabolites. The figure of the brain shows one of the three experimental groups, and the remaining two groups are illustrated in the accompanying figure (Figure S3).

In the experiment, we detected a total of 205 metabolites, of which 77 were identified. A total of 13 metabolites showed significant differences in different pathways. In the study of the energy metabolism pathway, we primarily analyzed the alterations in the lactic acid, glucose, fumaric acid, and malic acid levels (Figure 3A). Lactate levels exhibited a more uniform distribution throughout the entire brain and were significantly lower in the model group than in the control group. When considering the schematic diagram with these findings, it was evident that the decrease in lactic acid was predominantly concentrated in the anterior region of the mouse brain. The distribution of glucose in the mouse brain was naturally biased toward the posterior hemisphere; however, under the influence of CSDS, there was a significant accumulation of glucose near the cerebellum and striatum. The natural distribution of fumarate was homogeneous, whereas in the model group, a significant reduction in fumarate concentration was observed in the posterior brain regions. The malic acid concentration was greater in the posterior brain regions than in the anterior brain regions and showed a decreasing trend, particularly in the cerebellar cortex and accumbens-shell. In summary, the disparities in lactate levels were predominantly localized within the frontal region, whereas alterations in glucose, fumarate, and malic acid levels exhibited greater concentrations within the posterior region.

In the purine metabolism pathway, we primarily analyzed the alterations in AMP, inosine, hypoxanthine, deoxyguanosine, and uridine levels (Figure 3B). AMP exhibited a greater distribution within the cerebral cortex and a more balanced distribution in the remaining regions. We observed a decrease in the AMP concentration specifically within the striatum region. The distribution of inosine was predominantly observed in the Med, PN, CIC, and other regions. In the depression model group, elevated levels of inosine were detected specifically in the posterior brain regions and around the thalamus. Hypoxanthine and inosine are converted reciprocally in vivo. The distributions of hypoxanthine and inosine were very similar, and both were also upregulated in the depression model mice. The brain regions with the largest concentration differences occurred in the posterior region of the brain and around the thalamus. Deoxyguanosine was mainly distributed in the striatum and nucleus accumbens. Interestingly, when we analyzed deoxyguanosine, we found that the changes in different brain regions were opposite. The concentration of deoxyguanosine increased in the CPu, CBC, and Acbsh regions and decreased in the Med, CIC, and TH regions. These differences suggest that the synthesis and decomposition of deoxyguanosine may differ in different brain regions, and further analysis is needed. Uridine was mainly distributed in the posterior brain of mice, and the concentration of uridine increased more significantly in the hippocampus and medial prefrontal cortex in the model group. In general, there was a more significant metabolic upregulation of purine pyrimidine metabolism-related substances in the cerebellum, hippocampus, striatum, and surrounding regions.

In addition, we selected other substances that exhibited significant differences in the model for analysis. These substances included glutamine, aspartic acid, disodium phosphate, and potassium phosphate (Figure 3C). Glutamine was primarily distributed around the thalamus, and its concentration was increased in the model group. After the data analysis, concentration differences were found to predominantly occur in and around the striatum. Aspartate was mainly distributed in the Med, DpMe, CIC, and MPFC regions. In the depression model, the concentration of aspartate was significantly lower in the TH region. Disodium phosphate and dipotassium phosphate levels reflect changes in phosphoric acid levels within mouse brains. The concentrations of the two phosphates in various brain regions did not significantly differ. Both phosphates increased within the mouse brain. These findings revealed notable variations in the cerebellar regions and thalamus, as well as in the striatum regions and their adjacent areas.

The 13 metabolites were confirmed using standards (Figure 3D and Table S1). To better simulate substance detection in the mouse brain slices, standard solutions were mixed with mouse brain homogenates. Simultaneous sampling of different standards and mouse brain homogenates was conducted to validate that the signal originated solely from the added standard. The results verified that AMP, inosine, hypoxanthine, lactic acid, fumaric acid, malic acid, glutamine, and aspartic acid exhibited [M − H]^−^ negative ions. Na_2_HPO_4_ and K_2_HPO_4_ displayed metal-addition positive ions. Glucose, deoxyguanosine, and uridine produced [M + Cl]^−^ negative ions.

In summary, our study revealed and validated that the most prominent alterations in the three major substance categories and 13 metabolites were observed in the striatum and surrounding regions, hippocampus, medial prefrontal cortex, and cerebellum.

### 3.3. Areas of Significant Metabolic Change

**Figure 4:**
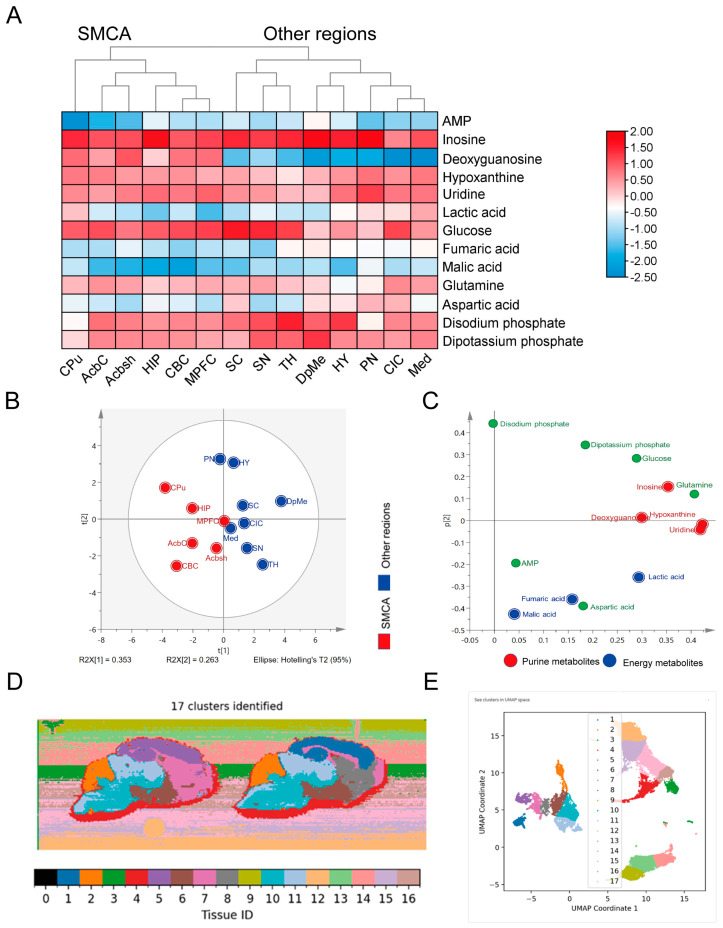
Regional salience of the significant metabolic change area (SMCA) and validation of this classification approach. (**A**) Clustering heatmap of brain regions and metabolites. The data were transformed into ratios comparing the model group to the control group, where red indicates an elevated concentration in the disease model, and blue represents a reduced concentration. The data were normalized to eliminate interference from material detection. The row labels list the metabolites; the top column labels indicate the class; the bottom column labels indicate the brain regions. (**B**) PCA score scatter plot of brain regions. (**C**) Loading scatter plot of brain regions. Red represents purine metabolites, while blue indicates energy-related metabolites. (**D**) UMAP analysis revealing metabolic variations across distinct brain regions. (**E**) Classification clustering graph generated by UMAP.

The differences between the model and control groups were transformed into ratios and visualized using a clustered heatmap (Figure 4A). The cluster analysis revealed that the brain regions could be categorized into two groups: CPu, HIP, AcbC, Acbsh, CBC, and MPFC as one category and Med, PN, CIC, SC, SN, TH, DpMe, and HY as another category. The disparities observed in the first group were significant and possessed distinctive attributes. For instance, several substances, such as deoxyguanosine and malic acid, exhibited noteworthy variations. After considering these data, we defined the brain regions in the first category as the significant metabolic change area (SMCA). Moreover, we observed a stronger correlation of the metabolic changes between the cerebellar cortex and cerebral cortex. Additionally, we noted a consistent pattern of metabolic changes between the nucleus accumbens core region and its adjacent shell region, supporting their close association.

To validate the reliability of the classification results, we employed SIMCA software for unsupervised data dimensionality reduction to examine the correlations among brain regions (Figure 4B). The unsupervised classification results showed that the CPu, HIP, AcbC, Acbsh, CBC, and MPFC brain regions were clustered in the lower left of the image during dimensionality reduction. Further analysis of their contributions revealed that energy-metabolism-related substances, such as lactic acid, malic acid and fumaric acid, and purine metabolism pathway substances, including uracil, inosine, hypoxanthine, and deoxyguanosine, contributed to two directions of discrimination (Figure 4C). Moreover, most substances were better distinguished in two directions of discrimination. This classification method is robust and reliable.

To further validate the brain regions affected by depression, we did not confine ourselves to the primary differential substances but instead utilized all the detected metabolite data to conduct uniform manifold approximation and projection (UMAP) dimensionality reduction. The UMAP dimensionality reduction data revealed that the mouse brain tissue could be segregated into distinct regions based on substance differences (Figure 4D). Although the classifications of most brain regions, such as the cerebellum and thalamus, were consistent between the CSDS model group and the control group, indicating minimal alterations in regional composition, notable differences in matter were observed within the cerebral cortex, hippocampus, and striatum nucleus accumbens. Consequently, these regions exhibited distinct clustering patterns. This finding underscores the substantial alterations in the composition of the SMCA region induced by the depression model, surpassing its inherent compositional characteristics. Additionally, we corroborated the reliability of this outcome. In Figure 4E, the left area corresponds to the mouse brain sample region, while the right area represents slide artefacts and other interferences. These findings confirmed that the classification of the mouse brain regions by UMAP in this study was reliable.

### 3.4. Common Features and Differences between SMCAs

**Figure 5:**
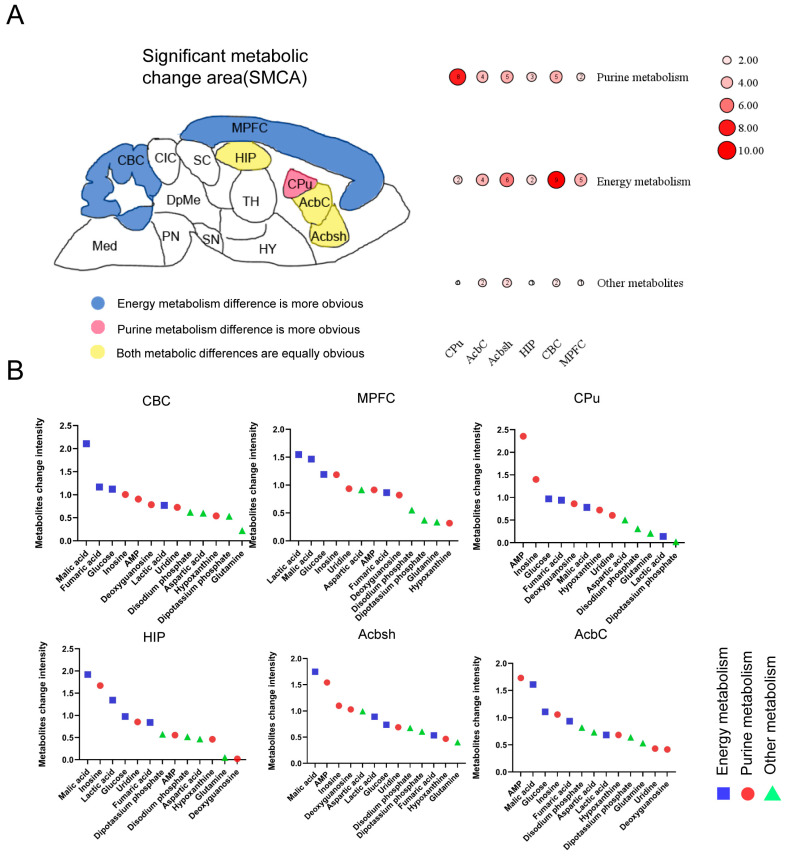
Common and differential features of metabolism within the SMCAs. (**A**) Significance map of metabolic pathways in the SMCA brain regions. The metabolites were classified according to their metabolic pathways. The fold changes were summarized and quantified into ten grades. (**B**) Metabolite sequencing diagram across distinct brain regions.

Simultaneous alterations in purine metabolism and energy metabolism within the SMCA were the primary characteristics of the CSDS depression model. However, there were variations in the significance of the alterations in energy metabolism and purine metabolism within the SMCA. Therefore, we further synthesized the shared characteristics and relative distinctions among the SMCA brain regions, thereby uncovering potentially broader associations within these brain regions. The disparities among the three metabolic types in the SMCA are shown in Figure 5A. The data revealed that energy metabolism discrepancies were more pronounced in the medial prefrontal cortex and cerebellar cortex. In the hippocampus, accumbens-core, and accumbens-shell, differences in both energy metabolism and purine metabolism were equally significant. Notably, purine metabolism variations were more prominent in the caudate putamen. Additionally, we arranged the metabolites according to the magnitude of the metabolic differences and classified them according to their respective metabolic pathways (Figure 5B). The three compounds exhibiting the most pronounced metabolic disparities in the medial prefrontal cortex and cerebellar cortex were all energy-related metabolites, whereas the two compounds displaying the greatest differences in the caudate putamen were purine metabolites. In the other brain regions, these two types of metabolites demonstrated a cross-distribution pattern. These findings provide a more comprehensive understanding of the metabolic alterations across distinct brain areas.

## 4. Discussion

Patients with depression may experience side effects such as headache and drowsiness when taking drugs that directly regulate neurotransmitters [34], due in part to the nonspecific targeting of these drugs, which increases the likelihood of adverse reactions. However, as mentioned in the Introduction, the specific brain regions and metabolic processes targeted by antidepressant drugs during development remain unclear. Therefore, an additional tool is required to simultaneously detect the metabolites in the various brain regions of the nervous system and provide guidance.

We innovatively employed a lipid-based brain region localization method. First, we observed that this approach exhibited superior universality, as lipids were detected in all mouse brain samples, and demonstrated good repeatability. Second, we observed that various lipid boundaries exhibited consistent repeatability and complete overlap. This implies the existence of an inherent barrier within the mouse brain that facilitates the classification of distinct brain regions and impedes unrestricted diffusion of metabolites across tissues. This phenomenon may arise from the distinct lipid composition ratio of different brain cells. Third, because the lipid distribution was more closely aligned with the natural distribution of endogenous metabolites, in situ lipid classification is a more suitable approach than traditional methods, such as HE staining analysis or UMAP dimensionality reduction, for investigating the variations in metabolic markers among different the brain regions of mice. Moreover, this classification approach offers greater accuracy and greater physiological significance.

The functional/bioinformatic methodology for mass spectrometry imaging is continuously advancing [35,36]. We employed the uniform manifold approximation and projection (UMAP) approach to validate the SMCA. UMAP, an effective approach for analyzing high-dimensional data, enables reliable and meaningful analysis while facilitating data visualization and interpretation [37]. It is widely utilized in mass spectrometry imaging to differentiate various cell types and aid in pathological assessment [38,39]. To accurately determine the differences between substances, data from both the control group and the model group were simultaneously collected in our experiment. Consequently, when employing UMAP dimensionality reduction analysis, a classification map of brain regions for both groups was generated on a unified platform. Comprehensive data analysis revealed significant differences in all SMCA regions except for the cerebellar region, confirming that the 13 characteristic metabolites effectively captured overall distinctions and validating the accuracy of the SMCA from an additional perspective.

Studies have demonstrated the therapeutic effects of exogenous malic acid supplementation on depression [40]. Our findings indicate reductions in fumarate and malic acid levels, both of which are important metabolites in the tricarboxylic acid cycle in the SMCA of CSDS mice, suggesting that energy metabolism dysfunction potentially underlies the pathogenesis of depression. Our experimental findings demonstrated an increase in glucose concentrations within the SMCA, accompanied by a concurrent decrease in lactate concentrations. The brain, which is highly energy-dependent, constitutes merely 2% of the body weight but accounts for 20% of oxygen consumption and 25% of glucose metabolism [41]. Depression significantly diminishes glucose metabolism and utilization within the MPFC [42], potentially leading to reduced lactate production. Emerging evidence suggests that lactate serves as a crucial energy substrate for the brain [43], with neurons exhibiting a preference for its utilization over glucose, while astrocytes predominantly metabolize glucose and release lactate [41]. Impaired astrocyte metabolism may cause dysfunction by inhibiting mitochondrial ATP production, thereby inducing depression-like behavior in animal models [44]. The reduction in and apoptosis of astrocytes are considered key mechanisms underlying depression [45,46,47], which is supported by our investigations into energy metabolism. Interestingly, our study revealed a more pronounced manifestation of energy metabolism dysfunction in the MPFC and CBC. Previous studies demonstrated a greater density of astrocytes in these two brain regions [48], which may serve as an intrinsic factor contributing to the co-occurrence of energy metabolism disorders.

Our previous work demonstrated that, compared with healthy control mice, CSDS mice exhibit significant increases in inosine, hypoxanthine, and uridine levels in the hippocampal region [49]. In this DESI-MSI experiment, we found that these alterations were prevalent not only in the hippocampal region but also extended to the SMCA. Interestingly, we noted an increase in deoxyguanosine levels specifically within the SMCA while observing a decrease in the surrounding brain regions. These findings suggest a potential role for deoxyguanosine in depression pathogenesis. In the ab initio synthesis pathway of purines, IMP serves as an initial precursor that is subsequently converted into substances such as AMP and GMP [50]. Among the purine metabolites, the SMCA exhibited elevated levels of inosine, hypoxanthine, uridine, and deoxyguanosine, indicating enhanced nucleotide synthesis activity. However, because certain factors inhibiting replenishment processes for AMP production within SMCA cells are limited, these limitations may play a crucial role in understanding depression-related mechanisms. The upregulation of purine metabolism is indicative of genetic material damage in cells, and studies have demonstrated that the most prominent characteristic of depression associated with the caudate putamen is a reduction in volume [51], which suggests that the caudate putamen is more closely linked to the upregulation of purine metabolism.

The absence of an integrated urea cycle in the central nervous system (CNS) means that glutamine synthesis is the sole mechanism that effectively counteracts blood ammonia levels or recycles intracellularly produced ammonia in CNS cells [52,53]. Glutamine accumulation is frequently observed in individuals with depression [17], and our experiments revealed that this accumulation was most pronounced in the nucleus accumbens region. Excessive glutamine buildup within brain cells may have detrimental effects on brain function primarily due to its interference with mitochondrial function and partly due to its osmotic effects [54]. We selected both dipotassium phosphate and disodium phosphate for the characterization of brain phosphate content. In conventional LC-MS detection, sample processing and protein precipitation may impact phosphate levels. Therefore, DESI-MSI enables better observation of the phosphate content and status. Inorganic phosphate in the brain is closely associated with energy metabolism [55,56]. For instance, mitochondrial oxidative phosphorylation efficiently catalyzes ADP and inorganic phosphate to generate ATP using energy derived from glucose metabolism. Phosphorylation also serves as a vital mechanism for delivering energy to various substances. We observed a widespread increase in phosphate levels, which is consistent with the widespread reduction in ATP levels observed during depression [57,58]. These findings further substantiate the aberrant nature of energy metabolism.

The application of mass spectrometry imaging technology can aid in clinical treatment, such as through the identification of biomarkers for liver cancer [59] or the analysis of the metabolic characteristics of intrabody chemotherapy drugs [60]. DESI-MSI technology was employed to analyze the metabolic changes in the brain associated with depression. Our findings suggest that targeted drugs for the SMCA brain region may demonstrate enhanced efficacy and reduced side effects. Modulating abnormal purine metabolism in the caudate putamen or addressing energy metabolic disorders, such as glucose accumulation and lactate supply deficiency in the cortex, may help alleviate depressive symptoms more effectively. This offers valuable insights for the development and evaluation of clinical antidepressant drugs.

## 5. Conclusions

In this study, we employed an innovative lipid-based DESI-MSI analysis method with excellent repeatability and distinct boundaries to investigate the metabolites and metabolic pathways in the brains of depressed mice in situ. We identified a diverse range of metabolites and validated 13 key metabolites using standard substances. The results demonstrated the simultaneous occurrence of energy metabolism disorders and the upregulation of purine metabolism in the significant metabolic change area (SMCA), which represents the most significant characteristic of CSDS-induced depression in mice. The research findings presented in this paper collectively demonstrate a paradigm of brain metabolic changes associated with depression, contributing to a deeper understanding of depression and providing valuable insights for the development and evaluation of clinical antidepressant medications.

## Data Availability

Data are contained within the article or Supplementary Material.

## Supplementary Materials

The following supporting information can be downloaded at: https://www.mdpi.com/article/10.3390/metabo14050284/s1, Figure S1. Diagram depicting the process of identification and exclusion of isotope ions; Figure S2. Representative examples of the same metabolites exhibiting variations in composition due to the presence of diverse additional ions; Figure S3. The remaining two sets of DESI-MSI images; Table S1. Metabolite ions identified using standards.
