# Supplementary material for: Desorption Electrospray Ionization Mass Spectrometry Imaging Techniques Depict a Reprogramming of Energy and Purine Metabolism in the Core Brain Regions of Chronic Social Defeat Stress Mice
Source: Metabolites. 2024 May 15;14(5):284. doi: 10.3390/metabo14050284 (PMC11123228; doi:10.3390/metabo14050284)
Supplement: Supplementary file 1 [file metabolites-14-00284-s001.zip › metabolites-2997089-supplementary.pdf]

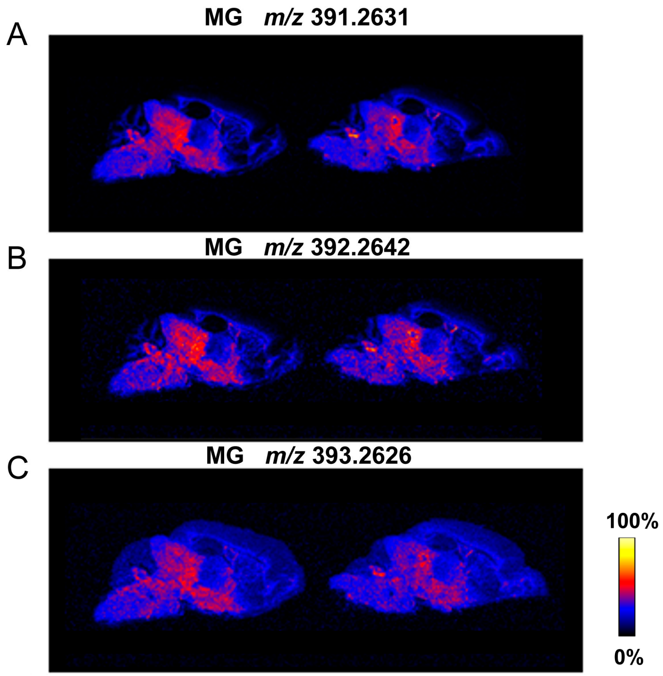

Figure S1. Diagram depicting the process of identification and exclusion of isotope ions.

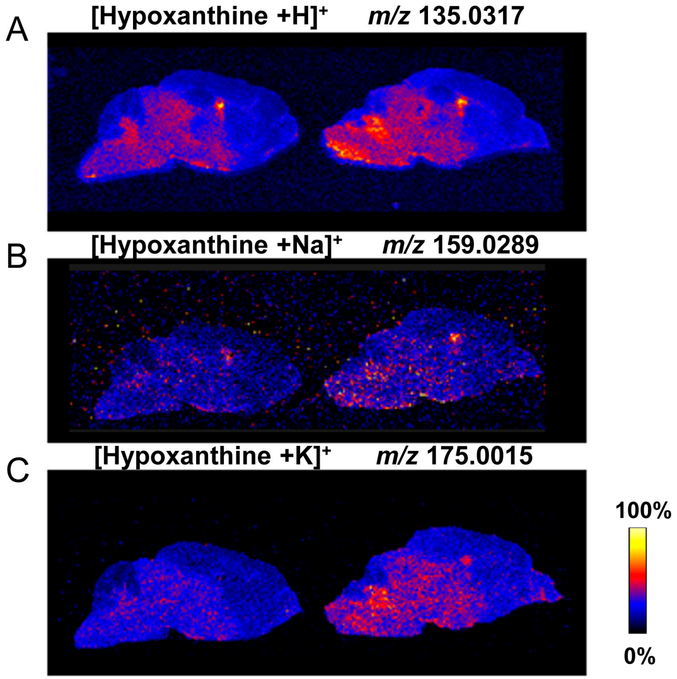

Figure S2. Representative examples of the same metabolites exhibiting variations in composition due to the presence of diverse additional ions. (A) [M +H]+ example. (B) [M +Na]+ example. (C) [M +K]+ example.

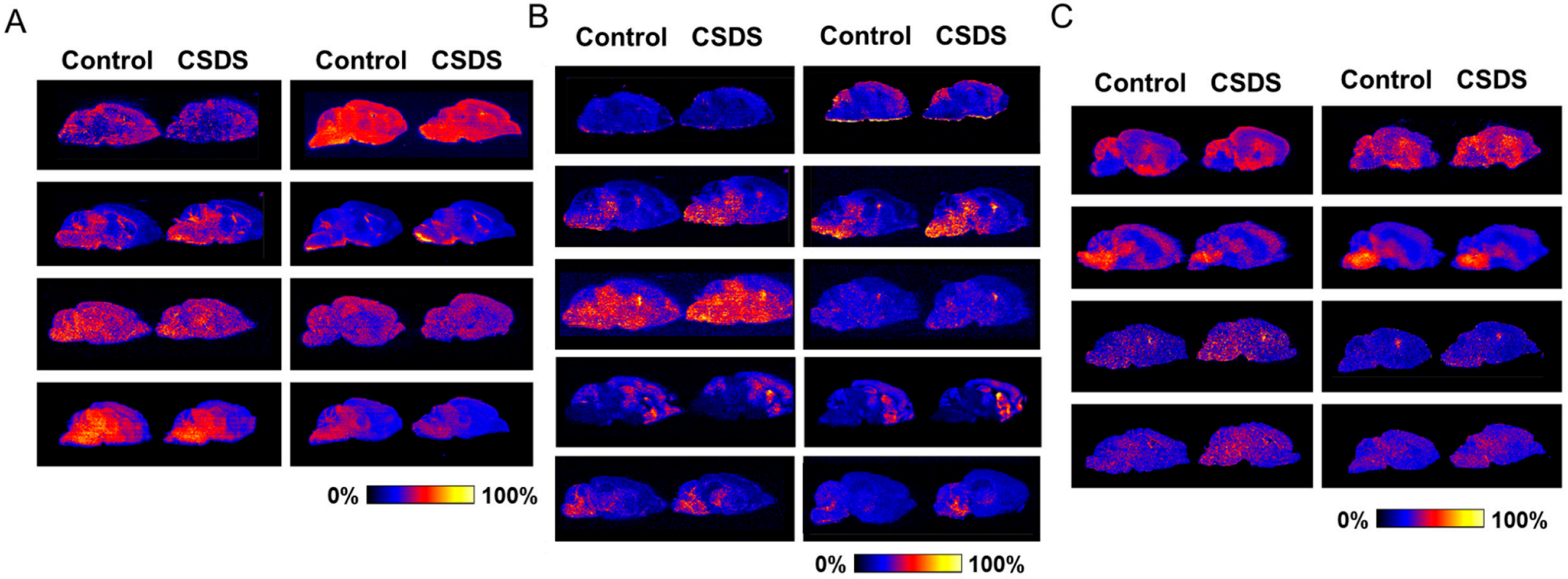

Figure S3. The remaining two sets of DESI-MS images. (A) Energy metabolism-related metabolite images. (B) Purine metabolism-related metabolite images. (C) Images of other metabolites.

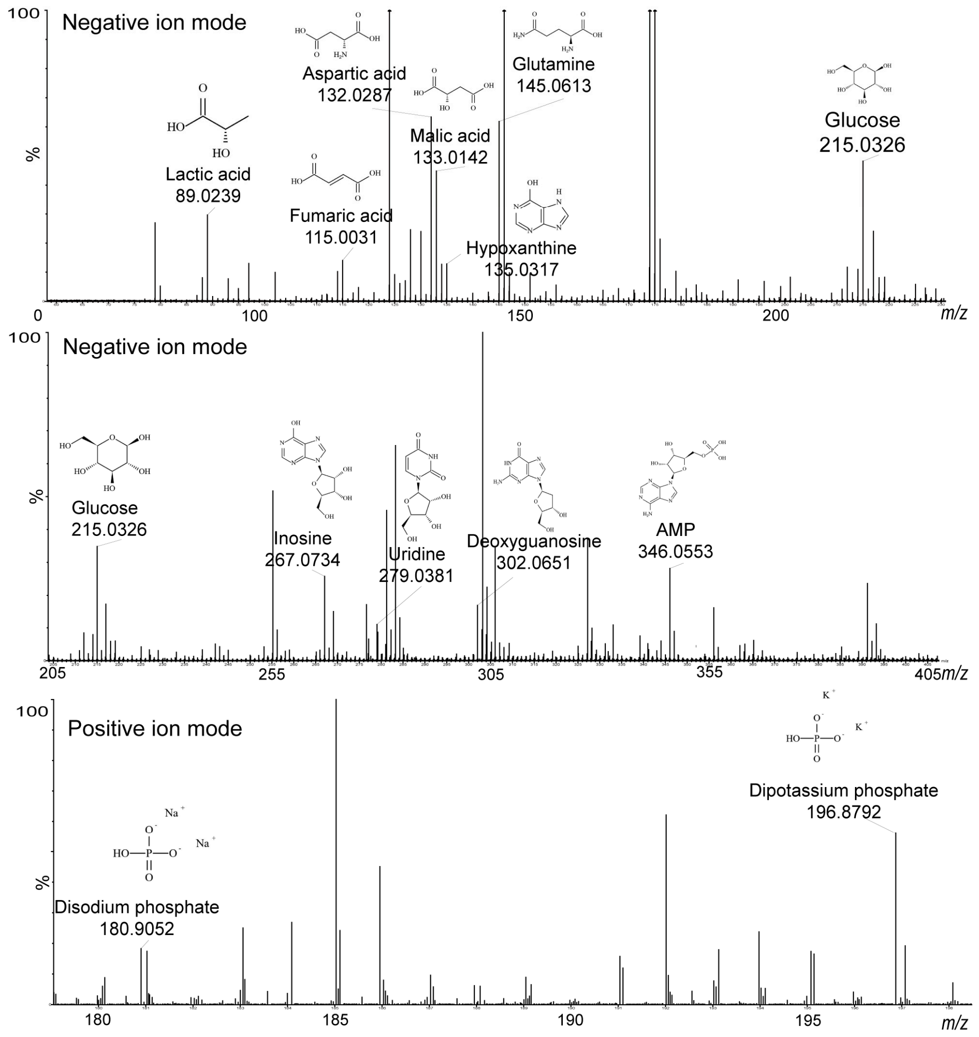

Figure S4. Mass Spectrum for Material Analysis.

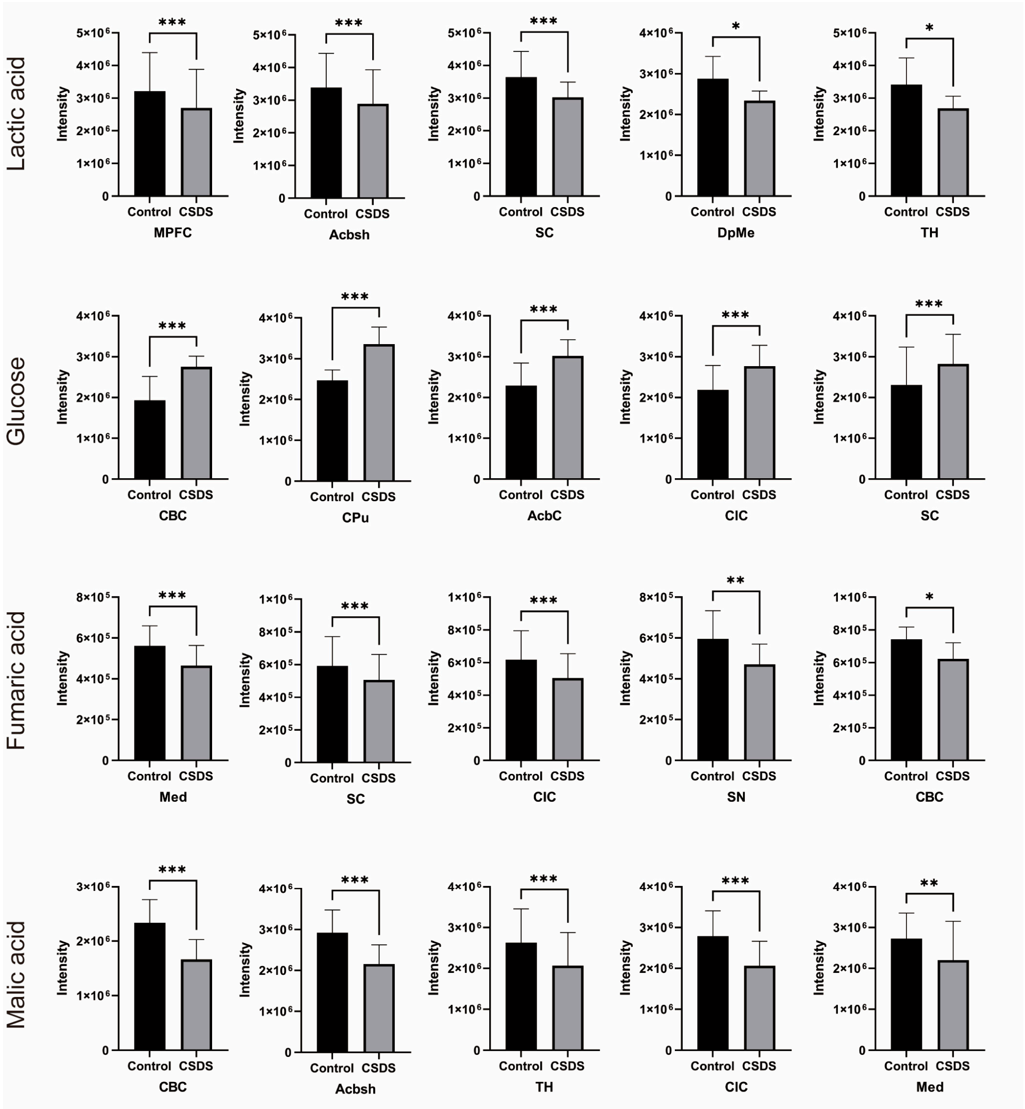

Figure S5. Regional significance analysis of energy metabolism-related substances. * $p < 0.05$, ** $p < 0.01$, *** $p < 0.001$.

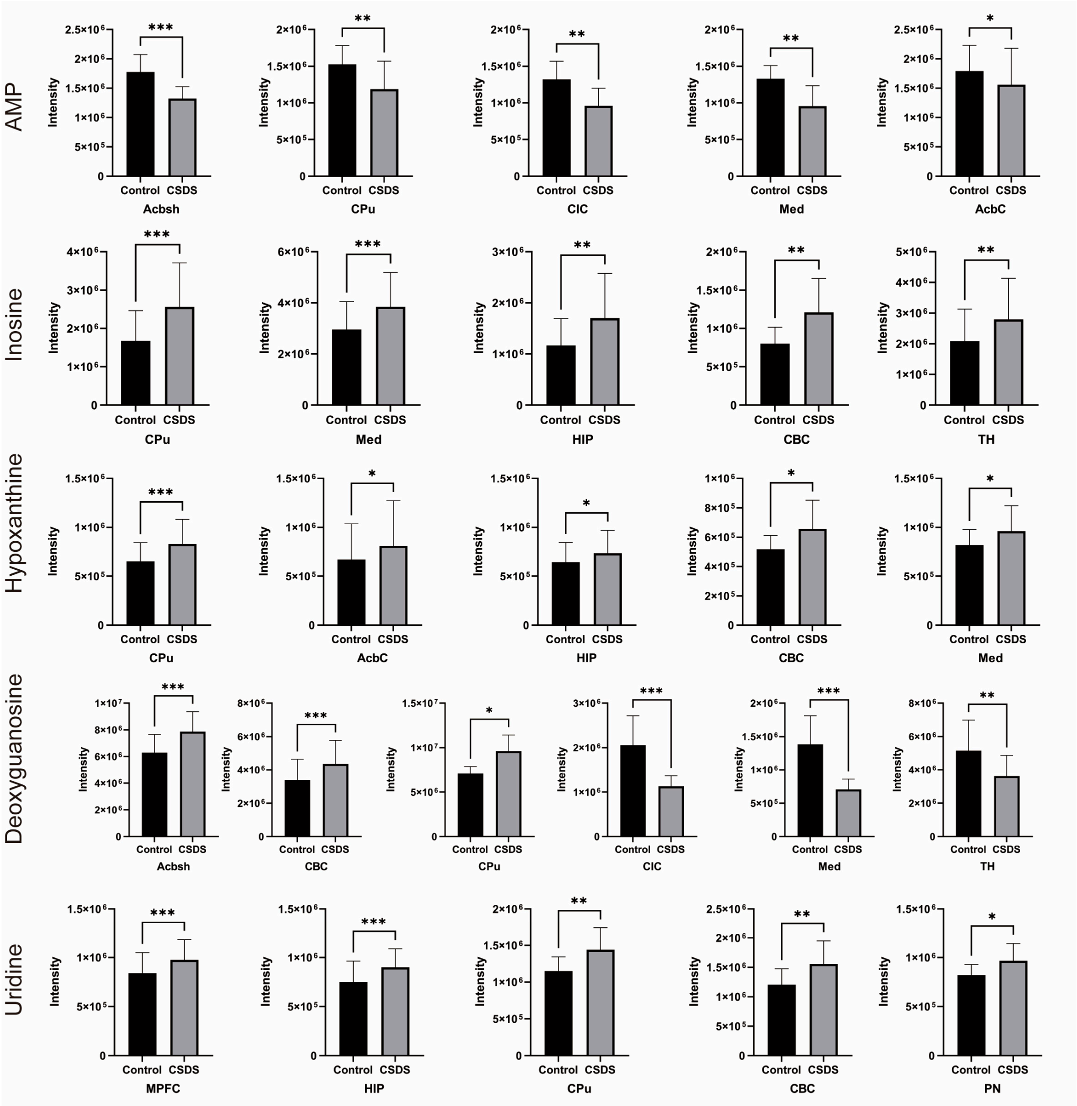

Figure S6. Regional significance analysis of purine metabolism-related substances. *$p < 0.05$, **$p < 0.01$, ***$p < 0.001$.

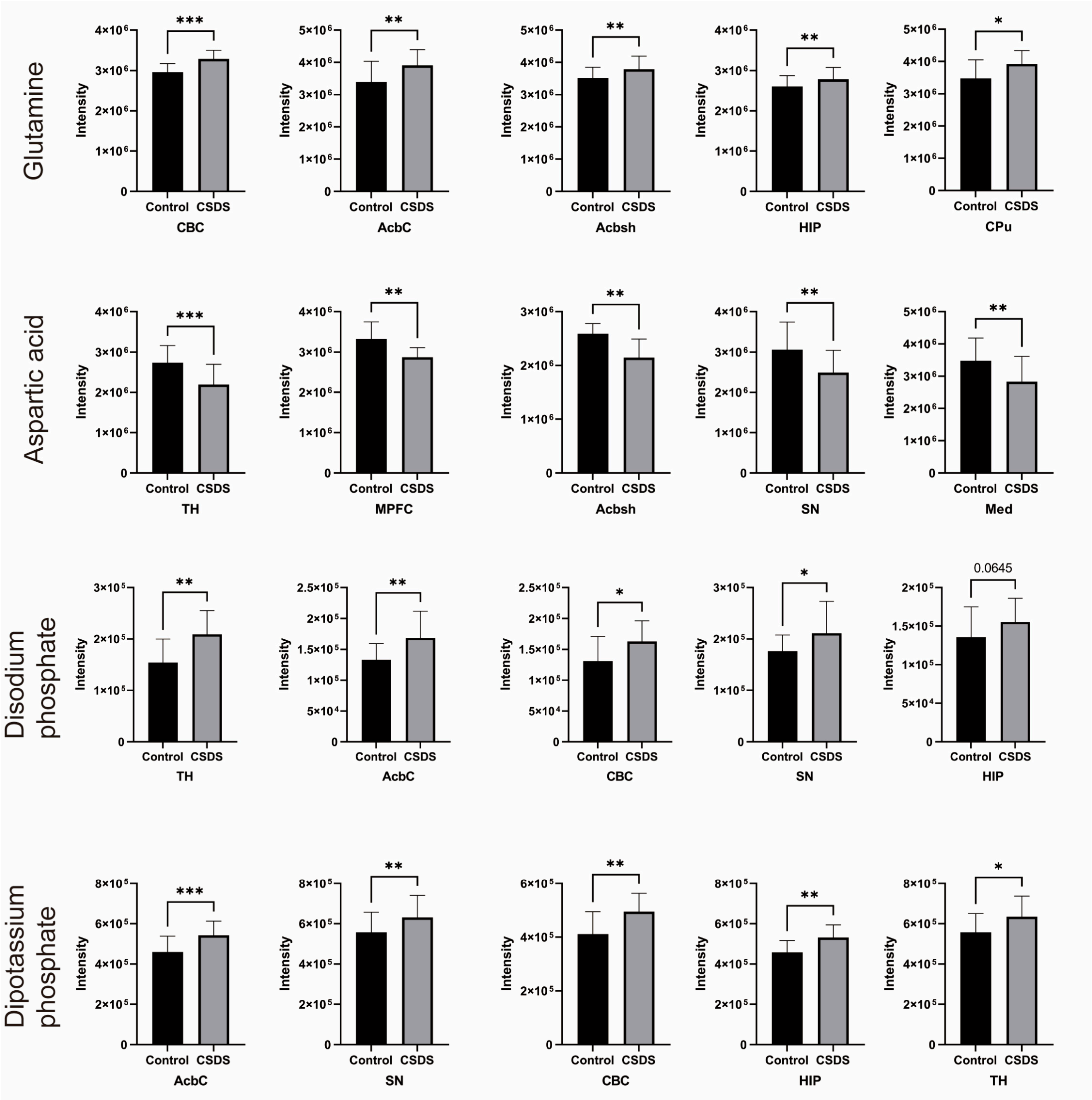

Figure S7. Regional significance analysis of other metabolism-related substances. *$p < 0.05$, **$p < 0.01$, ***$p < 0.001$.

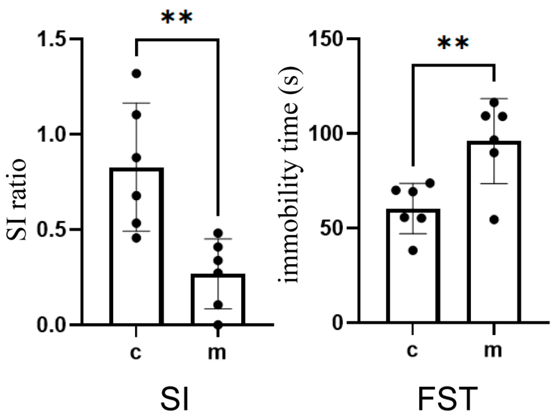

Figure S8. Behavioral testing of animals. $^{**}p < 0.01$.

Table S1. Metabolite ions identified using standards.

**Metabolites**	**ion type**	*m/z*	**Relative error（ppm）**
Lactic acid	$[M-H]^-$	89.0239	5.65
Fumaric acid	$[M-H]^-$	115.0031	4.93
Aspartic acid	$[M-H]^-$	132.0287	3.03
Malic acid	$[M-H]^-$	133.0142	0.24
Hypoxanthine	$[M-H]^-$	135.0317	3.56
Glutamine	$[M-H]^-$	145.0613	3.80
Inosine	$[M-H]^-$	267.0734	0.29
AMP	$[M-H]^-$	346.0553	1.42
Dipotassium phosphate	$[M+Na]^+$	196.8792	6.71
Disodium phosphate	$[M+K]^+$	180.9052	6.95
Glucose	$[M+Cl]^-$	215.0326	0.88
Uridine	$[M+Cl]^-$	279.0381	3.00
Deoxyguanosine	$[M+Cl]^-$	302.0651	3.49
